# Metabolic acidosis and sudden infant death syndrome: overlooked data provides insight into SIDS pathogenesis

**DOI:** 10.1007/s12519-024-00860-9

**Published:** 2024-12-10

**Authors:** Paul N. Goldwater, Dov Jordan Gebien

**Affiliations:** Adelaide Medical School, Faculty of Health and Medical Sciences, The University of Adelaide, North Terrace, Adelaide, South Australia 5006 Australia

**Keywords:** SIDS, Metabolic acidosis, Dyshomeostasis, Diaphragm, Heart

## Abstract

**Background:**

Decades of mainstream SIDS research based on the Triple Risk Model and neuropathological findings have failed to provide convincing evidence for a primary CNS-based mechanism behind putative secondary dyshomeostasis (respiratory or cardiac) or impaired arousal. Newly revealed data indicate that severe metabolic acidosis (and severe hyperkalemia) is a common accompaniment in SIDS. This supports the direct effect of sepsis on vital-organ function and occurrence of *secondary* CNS changes accompanied by the dyshomeostasis leading to SIDS.

**Data sources:**

Using PubMed and Google Scholar literature searches, this paper examines how metabolic acidosis and sepsis might contribute to the underlying pathophysiologic mechanisms in SIDS.

**Results:**

The discovery of a series of non-peer-reviewed publications provided the basis for a serious examination of the role of metabolic acidosis and sepsis in SIDS. Most SIDS risk factors relate directly or indirectly to infection. This consequently elevated the position of septic or superantigenic shock and viremia in causing secondary organ failure leading to SIDS. The latter could include diaphragmatic failure, as evidenced by peripheral respiratory (muscle) arrests in experimental septic shock, as well as infectious myositis and diaphragm myopathy in sudden unexpected deaths, including SIDS. In addition, just as acidosis lowers the threshold for ventricular fibrillation and sudden cardiac arrest, it could also contribute to similarly unstable diaphragm excitation states leading to respiratory failure.

**Conclusions:**

This paper uniquely reveals compelling evidence for a connection between metabolic acidosis, sepsis, viral infections, and sudden unexpected child deaths and provides a solid basis for further work to define which pathway (or pathways) lead to the tragedy of SIDS. It is recommended that all autopsies in sudden unexpected deaths should include pH, bicarbonate, lactate, and electrolyte measurements, as well as diaphragm histology.

## Introduction

**Fig. 1:**
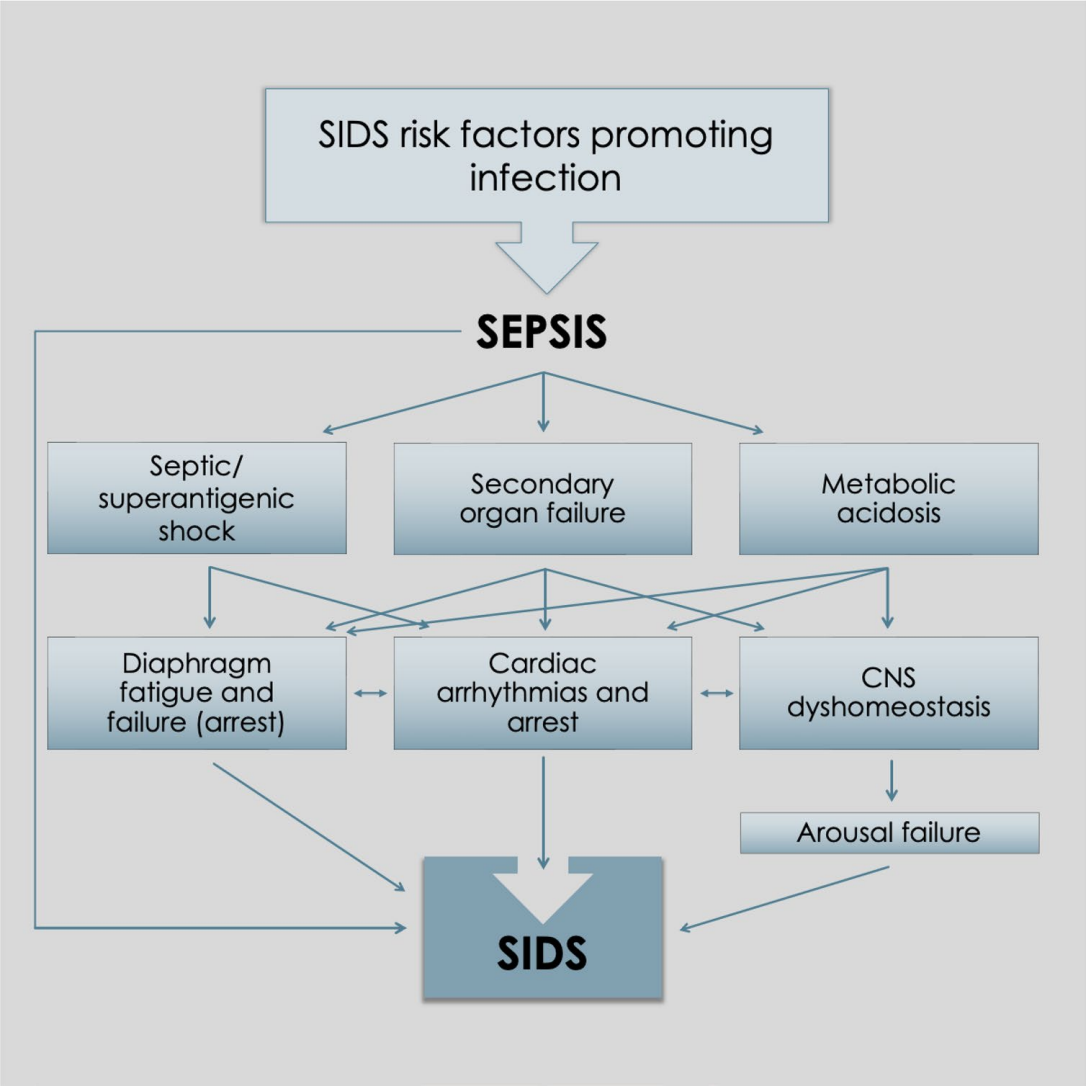
Proposed pathogenetic pathway leading to sudden infant death syndrome (SIDS)

Sudden infant death syndrome (SIDS) is defined as “the sudden death of an infant under 1 year of age which remains unexplained after thorough investigation including a complete autopsy, death scene investigation, and detailed clinical and pathological review” [1]. The prevalence of SIDS has declined in most developed countries from a high prevalence of 2 to 5 per 1000 live births in the 1980s to < 1 per 1000 live births currently. Developing countries have high rates of SIDS. Researchers have paid close attention to the model of SIDS, known as the triple risk hypothesis [2]. It has undergone several iterations over the decades and has been used to form a basis for scientific research. The hypothesis supposes that SIDS results from a lethal combination of the three major influences: general vulnerability, age-specific risks, and precipitating stressors. The risk factors for SIDS are listed in Table 1. Notably, these generally parallel the risk factors of susceptibility and/or infection. The triple risk hypothesis can be applied equally to most infectious diseases. Despite the connection with infection, mainstream SIDS researchers have focused their energy on homeostatic control of breathing, arousal and cardiac function, with the prone sleep position risk factor used as the guiding factor in this complex condition. There are characteristic pathological and laboratory findings in cases of SIDS that are commonly observed. Characteristic gross pathology includes intrathoracic petechial hemorrhages, liquid-unclotted heart chamber blood, and organ weight abnormalities (heavy brain, thymus, liver, and lungs, and lighter hearts) [3]. The laboratory findings in SIDS are listed in Table 2. A majority tend to reflect a possible underlying infection or immunopathological process (Fig. 1)..

**Table 1:** Risk factors for sudden infant death syndrome

Factors
Ethnicity
Male sex
Developmental
Prematurity/intrauterine growth retardation
Peak age range 2–4 mon
Prenatal/pregnancy
Higher parity
Low birth weight, short gestation (intrauterine growth retardation)
Inadequate prenatal care
Maternal smoking
Night time
Environmental
Mild infections (URTI or gastroenteritis) (recent illness potentiates effect of prone sleep position and overwrapping)
Recent visit to general practitioner or outpatient clinic
Prone sleeping
Cigarette smoke exposure
Overheating
Cooler season
Lack of breastfeeding
Poor socio-economic conditions
No or late immunisation
Air pollution
Contaminated sleeping surface: used cot mattress, sofa, parental bed
Day care attendance
High birth order/older siblings

Original information can be found in Ref. [5]. *SIDS* sudden infant death syndrome, *URTI* upper respiratory tract infection

**Table 2:** Laboratory findings in SIDS cases

Laboratory findings
Mild acute inflammatory changes in airways, lungs, and myocardium
Central nervous system inflammatory reaction seen with microglial activation and astrocytosis and neuronal apoptosis
Proteomic and immunohistochemical evidence of infection and responses to infection, bacterial toxins in tissues
IgG response to bacterial toxins
Increased IgM response to core endotoxin
Increased levels of mast cell tryptase
Increased levels of mannose-binding lectin
Raised fibrin degradation products
CD68 immunoreactivity in airways and brain
Raised IL-6 in vitreous humour, cerebrospinal fluid, and liver
CSF lymphocytosis
Presence of tracheal/lung IgM
IL-10 low producer
IL-1b high producer
Raised IgA in duodenum and saliva
Normally sterile site cultures yielding a bacterial pathogen

Original information can be found in Ref. [5]. *SIDS* sudden infant death syndrome, *IgG* immunoglobulin G, *IgM* immunoglobulin M, *IL* interleukin, *CSF* cerebral spinal fluid, *IgA* immunoglobulin A

Mainstream SIDS researchers’ focus on central nervous system (CNS) homeostatic control of breathing, arousal and cardiac function has provided a plethora of findings covering several decades of investigation. These findings are related mainly to a variety of suspected neuropathological and neurochemical changes within the CNS but with little ability to guide researchers as to whether the changes are primary or secondary in nature. These findings rarely correlate with epidemiological risk factors. Despite the decades-old knowledge that inflammatory cytokines are found in the CNS of SIDS cases [3], it has not until recently been demonstrated that viral infection [4] could be responsible, and that this pathology occurs as a secondary phenomenon (not primary, as purported in the triple risk model). Mention of metabolic acidosis in the SIDS research literature is largely missing or perfunctory. Infection and sepsis (with concomitant fluid losses and tissue hypoperfusion) are the leading causes of metabolic acidosis in infants and given that the risk factors for SIDS, almost without exception, point to infection being involved, the lack of research into metabolic acidosis is opprobrious [5]. This paper returns to and re-examines overlooked important data and provides a unique insight into the origin and effects of metabolic acidosis in SIDS pathogenesis. It may provide an opportunity to expand our understanding of the processes that lead to the tragic sudden unexpected deaths of human babies.

## New findings of metabolic acidosis in sudden infant death syndrome

Researchers often do not come across findings that illuminate a productive research path (in contrast to that followed for so long by most mainstream researchers). The findings, which were reported in two abstracts and in a 2006 Medscape article, but not as a paper in a peer-reviewed journal, are the work of the late Hazel L. McGaffey, an experienced pathologist based in Idaho, USA. In the first abstract [6], Dr. McGaffey referred to 12 SIDS victims, whereas in the second [7],15 consecutive unembalmed cases. The 12 cases were gathered over a 3-year period. Specific chemical, serologic, bacteriologic, hematologic, and viral studies were conducted, with five cases studied for blood pH and electrolytes obtained 3–10 h postmortem. Anatomic and histologic findings were found to be unrewarding; however, some intriguing clinical laboratory results were reported. Electrolytes and pH were compared between SIDS cases and infants who had died from other causes. Electrolyte levels (and rates of change) at various postmortem intervals in adults were also performed). In 1970, the incidence of sudden unexplained deaths in infants in Idaho Falls County, compared with live births, was higher than that of any other cause of death in this age group. pH and electrolytes were notable for hyponatremia, hyperkalemia, hypercalcemia, hyperphosphatemia and severe chronic acidemia. Normal blood urea nitrogen and “normal hydration” were detected. McGaffey postulated that these infants were in severe chronic electrolyte and pH imbalance with acidosis and that some added stress or cause for increased acidosis had occurred, resulting in death (such as upper respiratory infection, aspiration, or the “added acidemia of sleep”). Speculation as to the cause of these abnormalities, with reference to concomitant thinning of the adrenal cortex (hypoaldosteronism) and parathyroid gland findings, was also made.

McGaffey continued to work on this problem, as recorded in an article published in *Medscape* in 2006 by MacReady [8], who reported on McGaffey’s oral presentation at the American Society for Clinical Pathology meeting at Las Vegas in 2006. The work was published in the *American Journal of Clinical Pathology* in 2006 [9]; however, the reference was unable to be found in the online journal version despite being cited by Deixler [10]. Nevertheless, the *Medscape* article provided very valuable information that could lead to a better understanding of SIDS pathogenesis.

Several assumptions are made in interpreting Dr. McGaffey’s findings, including the cases being defined as SIDS. The data of the oral presentation, documented in the *Medscape* report, challenge the notion that respiratory acidosis is the etiology of most, if not all, SIDS cases. Rather, McGaffey’s findings suggested that severe metabolic acidosis underlies SIDS. Between 1965 and 1987, as a coroner/pathologist, she conducted laboratory studies on SIDS cases. Venous blood drawn from the superior sagittal sinus and cerebrospinal fluid drawn from the *cisterna cerebellomedullaris* were analyzed in 40 SIDS patients who had died between 1 and 8 months of age. Samples were collected at an average of 7 h postmortem. The findings were compared with children and adults who had died from other causes (number not stated), including respiratory and cardiac illness, acute trauma and various chronic diseases. The SIDS cases demonstrated extreme acidosis, with an average pH of 6.15, whereas it was 6.65 among children who died from respiratory causes. The SIDS bicarbonate concentration (base excess) decreased to an average of 6.31 mEq/L compared with an average of 15.8 mEq/L among cases of respiratory death.

Dr. McGaffey speculated that brainstem respiratory center shutdown is secondary to severe metabolic acidosis, with an increase in carbonic acid levels (averaging 5.24 mEq/L in SIDS cases compared with 2.33 mEq/L in respiratory deaths) suggesting that metabolic acidosis was the cause of death, whereas respiratory acidosis is associated with lower, not higher, carbonic acid. Severe disturbances in electrolytes were also found, with extreme hyperkalemia being a striking finding. SIDS babies had an average potassium concentration of 24.4 mEq/L compared with a normal range of 4.1–5.3 mEq/L. They also had hyponatremia, with an average sodium level of 127.9 mEq/L compared with a normal range of 139–146 mEq/L. Anoxia was suggested by a lactic acid level of 22.6 mmol/L and the presence of nucleated red blood cells. Elevated uric acid and blood urea nitrogen levels demonstrated early renal failure. McGaffey speculated that metabolic acidosis develops several days before death, citing a case of a 2-month-old male admitted with a low-grade fever and croupy cough. His pH was 7.2, and the HCO_3_^−^ concentration was 16.52 mEq/L. Potassium was elevated at 6.1 mEq/L, with anemia and relative lymphocytosis present, among other findings. He was given penicillin, improved, and was discharged, but symptoms returned. About 10 days later, he was found dead in his crib. A full autopsy, including postmortem clinical laboratory testing, revealed findings “typical of SIDS” at 7–8 days after the original tests. McGaffey suggested that the development of metabolic acidosis in SIDS may take considerable time and if it is noticed and treated, it could prevent death [7–9].

Although Dr. McGaffey’s findings reflected processes in only 40 cases of “presumed” SIDS, this data provides SIDS researchers with a new platform to evoke reconsideration or reinforcement of old paradigms (especially the common bacterial toxins hypothesis). In the meantime, it is recommended that full autopsies always include measurements of pH, bicarbonate, lactate, and electrolytes in venous blood drawn from the superior sagittal sinus as well as cerebrospinal fluid from the cisterna cerebellomedullaris. Doing so would be useful to corroborate or refute McGaffey’s findings. Moreover, efforts to investigate the lethal toxicity of SIDS sera should be strongly considered as part of future SIDS investigations (vide infra).

The discovery of Dr. McGaffey’s work led us to explore other studies examining pH and lactate level in SIDS, which revealed the work of Butterworth and Tennant (1989) [11]. They reported important pH and lactate differences in the brains of SIDS victims compared to those of control infants and control adults (as measured in the frontal and temporal cortices, cingulate gyrus, and caudate nucleus). Low lactate and high pH values were observed in sudden adult deaths (myocardial infarcts). Agonal-state cases had high lactate and low pH. Control infants (sudden traumatic deaths by accidents) had low lactate and high pH, whereas infants who were possibly hypoxic before death had high lactate and low pH. The results of the SIDS cases were divided into two groups: Group 1 included roughly one-third to one-half of those under 30 weeks of age who had low pH and high lactate levels. Group 2 included all those over 30 weeks of age and roughly one-half to two-thirds of those under 30 weeks with high pH and low lactate. The differences in lactate levels and pH values indicated that most SIDS cases had died suddenly, with a minority exposed to hypoxia just prior to death (from agonal breathing against airway obstruction). In addition, lactate levels and pH were significantly correlated across the four CNS areas, whereas lactate and pH were significantly correlated within each brain area, with a pH of 7.2 for zero lactate. Heart blood lactate was significantly correlated with brain lactate.

Among the sparse literature on acidosis in SIDS is a case report by Kinney et al. [12], which described severe metabolic acidosis with increased anion gap and lactate elevation in a previously healthy 8-month-old boy who suddenly died following seizures and respiratory distress. Inborn errors of metabolism were ruled out. A low perfusion state was thought to have caused lactic acidosis; however, the seizure itself could have been responsible (by convulsive muscle activity with consequent shift to anaerobic metabolism). Mild focal bronchopneumonia was present at autopsy, but its microbiology was not reported. Moon et al. [13] briefly mentioned metabolic acidosis in the context of the triple risk hypothesis, citing Kinney 2009 [14], in that it may contribute to other factors leading to SIDS deaths, including ineffectual gasping, progressive asphyxia, overheating and hypotension.

If the findings by McGaffey, Butterworth & Tennant and the abovementioned papers are confirmed, then metabolic acidosis could be used as a central starting point for researchers to develop several different theories of SIDS causation. Inborn errors of metabolism have been well investigated and do not account for more than a tiny minority of cases [15, 16]. In terms of more plausible causes, these could include the following:

1. Sepsis (including bacterial toxemia, toxic shock, superantigenic shock, and viral sepsis syndrome);
2. Sepsis with secondary vital-organ failure [including diaphragm-based respiratory failure, CNS-based respiratory failure, cardiac arrhythmias/asystole with increased genetically predisposed risk, genetically influenced mitochondrial energy failure (inborn errors of metabolism)].

Each of the above possible pathways to SIDS is discussed below (summarized in Fig. 1), with the exception of genetic conditions/inborn errors of metabolism.

## Sepsis

The plausibility of sepsis and vital-organ failure (cardiac arrest and diaphragmatic failure) is discussed below. However, genetically based cardiac arrhythmias/asystole, CNS-based respiratory failure and inborn errors of metabolism have been reviewed elsewhere and are not discussed in the paper.

### Infection, sepsis and toxemia

The reason why infection could be involved in the causal pathway to SIDS has been obvious since the first published reports of “cot death”, as it was previously known. Many early reports and studies have noted the common associations with symptoms and signs of viral respiratory infection [17–25].

Morris et al. [26] proposed the Common Bacterial Toxin Hypothesis in 1987. This was based on the frequent isolation of toxigenic bacteria (*Staphylococcus aureus, Streptococcus pyogenes* and *Escherichia coli*) from the nasopharynx of SIDS victims. A preceding viral respiratory infection stimulates mucus production and induces the accumulation of respiratory secretions through impaired mucociliary action, thus providing a medium for bacterial growth. Fever is another product of viral infection, also inducing bacterial toxin production. The hypothesis included the important role of the declining protective effect of transplacental maternal IgG, at SIDS peak ages of 2 – 4 months, and provided statistical support in this regard [26, 27].

The role of bacterial toxins in SIDS has been well established through multiple studies showing the lethal toxicity of sera obtained from SIDS victims. This lethal toxicity of sera and/or demonstration of toxins in sera and tissues from SIDS cases provides strong evidence^.^ [26–33]. However, despite such compelling findings pointing to the underlying cause of SIDS, it has been inexplicably ignored by mainstream researchers.

In addition, Highet et al. [34, 35] reported that *Staphylococcus aureus* and its enterotoxin genes are commonly found in SIDS cases. They demonstrated a highly statistically significant link between the risk factor of prone sleeping and the detection of *Staphylococcus aureus* in SIDS. Toxigenic bacteria other than *Staphylococcus aureus*, including *E. coli,* have also been found associated with SIDS [31, 36, 37].

The infectious hallmarks of SIDS include two major features: typically, nonlethal viral infections (both respiratory and gastrointestinal) and the presence of common toxigenic bacteria. This combination suggests that these could act in concert (alluded to previously): viral infection acts as a trigger for colonization, followed by superantigen toxin production culminating in superantigenic shock. To develop this idea, Nobel Laureate Peter Doherty and colleagues chose a mouse model and a murine respiratory virus (lymphocytic choriomeningitis virus, LCMV) together with staphylococcal enterotoxin-B (SEB). Mice infected with LCMV and injected intraperitoneally with SEB rapidly died of hematogenous shock. However, exposure to viruses or toxins alone was nonlethal [38].

Later important work indicating that viral infection could indeed act as a trigger for SIDS was provided by Harrison et al. in 1999 [39], wherein all the elements—viral infection, bacterial colonization, toxin induction, etc.—were effectively demonstrated. Recent findings by Ramachandran et al. [4], who used next-generation multiomic analysis of CNS tissue and cerebrospinal fluid, demonstrated the presence of viruses in the CNS of SIDS victims. These findings provide a new impetus for the long-held infection model of SIDS pathogenesis.

The epidemiology of infectious disease and that of SIDS largely overlap. Both are associated with poverty, poor hygiene, overcrowding, prematurity, exposure to tobacco smoke (postnatally and during pregnancy) and seasonality.

Sleeping prone on a secondhand mattress [40], parental bed [41] or sofa [42] increases the risk of SIDS through possible exposure to toxigenic bacteria contaminating those surfaces. Epidemiological studies have shown that prone sleep positioning is a significant risk factor for SIDS but only when the infant has a simultaneous respiratory or gastrointestinal tract infection [43, 44]. Uninfected babies sleeping prone remain at a similar risk to uninfected, supine-sleeping babies [43, 44]. Since the cardiorespiratory paradigm has not been shown to establish meaningful, broad and reproducible epidemiological concordance, mainstream research has not investigated the connection between prone sleep position and infection until only very recently [45, 46].

Male sex [47] and high birth order—in which infected older siblings potentially bring a virus home and expose the infant and, similarly, day care increasing the SIDS risk [48]—also align with infection. Seasonality is another feature of SIDS, with more cases occurring in the colder months in accordance with viral epidemiology [49–51].

The toxin findings, usually in a majority of SIDS cases together with the other epidemiological features, represent evidence for a process that eventuates in a septicemic/toxemic event. This could lead to profound metabolic acidosis terminating in a SIDS death directly, or indirectly, through the effect of acidosis on various vital organs. These indirect possibilities are discussed below.

## Sepsis with secondary organ failure

### Sepsis, diaphragmatic failure and lethal arrhythmias

Sepsis causes end organ damage through a multitude of biochemical processes, and skeletal muscles such as the limbs and diaphragm feature unexceptionally. Both become atrophic and weakened, resulting in reduced contractility and endurance [51, 52]. This is important because the diaphragm is the primary muscle of respiration. Diaphragm fatigue (or diaphragm dysfunction, DD), distinguished from weakness by its reversibility with rest, is common among intensive care unit (ICU) patients and can occur rapidly and profoundly. Acute bacterial infections can diminish diaphragm contractile strength by as much as 80% within 24 h of ICU admission [53]. Severe sepsis is often complicated by concomitant hypoxemia, hypovolemia, acidosis, and comorbid disease [54]. Evidence supporting peripheral, or type II, hypercapnic (ventilatory pump) failure in sepsis was first demonstrated in anesthetized dogs by Hussain et al. in 1985 [55]. As measured by transdiaphragmatic pressures, airflow rates and diaphragmatic electromyography, all animals given intravenous *Escherichia coli* endotoxin developed rapid respiratory insufficiency followed by tachypnea, bradypnea, and then sudden apnea leading to cardiac arrest within 1‒2 min. Death took only 150‒270 min. Hussain went on to declare that, in septic shock patients [ones not receiving mechanical ventilation], peripheral respiratory failure was the most important cause of death, even after hemodynamic aberrations had been corrected [56]. Sepsis-associated acute lung injury further contributed to the fatigue and failure by increasing ventilatory workloads.

Although the pathological mechanisms responsible for diaphragm weakness, fatigue, and failure in sepsis are complex and beyond the scope of this paper, they generally include activation of the proteolytic pathways as well as overproduction of inflammatory cytokines, prostaglandins, reactive oxygen species and nitric oxide. At the cellular level, this leads to structural myofiber injury (inducing organ atrophy and myopathy), impaired action potential generation and propagation through failure of neuromuscular and intradiaphragmatic depolarization, and disrupted excitation‒contraction coupling or damage to the contractile machinery itself. At the molecular level, contractile dysfunction occurs through altered Ca^2+^ homeostasis, which is influenced by endotoxins, acidosis, electrolyte disorders, and muscle fatigue itself, among other factors [57–59]. In experimental endotoxemic rats, reduced diaphragmatic contractility and endurance were associated with a decreased resting membrane potential and prolonged muscle relaxation time [54]. Although subsequent diaphragm neuromuscular inexcitability contributes to further fatigue [60], pathological excitation also occurs. Other than persistent hiccups, however, there is a paucity of data. A few case reports did reveal a variety of “hyperexcitation disorders”, including diaphragm spasms, tics, tremors, and myoclonus, as well as low- and high-frequency diaphragmatic flutter [61, 62]. Patients of all ages are affected by these apparently obscure disorders and generally present with chest or epigastric pain, sometimes with visible epigastric pulsations, and a variety of gastrointestinal and respiratory symptoms. The latter ranges from dyspnea and transient apneas in tetraplegic patients with diaphragm spasms [63], to respiratory distress and prolonged apneas requiring ventilatory support just hours after birth in neonates with respiratory flutter [62]. In addition, in a compelling pediatric case recently published, a sustained diaphragm spasm (akin to a tetanic cramp-contracture) was proposed to cause sudden unexpected respiratory arrests in children with severe diaphragm fatigue [64].

Like the heart, the diaphragm is a vital pump essential to life. Suddenly impaired cardiac output (cardiac arrest) from asystole or malignant cardiac arrhythmias is known to cause sudden unexpected deaths (for example, from ventricular fibrillation or various unstable ventricular tachycardias). Similarly, extremely rapid or ineffective diaphragm contractions which significantly impair lung alveolar ventilation could also lead to sudden deaths by respiratory failure. Sepsis, which is known to contribute to cardiac arrhythmias and arrests [65], is also suspected to impair diaphragmatic function through an analogous mechanism contributing to critical diaphragmatic failure in sudden infant deaths (i.e., unstable diaphragm arrhythmias) [64, 66]. In fact, DD (and work overload) is induced and exacerbated by several SIDS risk factors, including viral respiratory or gastrointestinal infections [diaphragm damage from myositis and myopathy (vide infra) with concomitant dehydration, acidosis, and electrolyte disorders], the young infancy period (harder working underdeveloped, untrained ventilatory muscles), rebreathing exhaled gases (causing net hypoxemia, hypercapnia, and respiratory acidosis; all worsening diaphragm contractility), tobacco smoke exposure, prone positioning, and rapid eye movement (REM) sleep (CNS inhibition of airway dilator and accessory respiratory muscles) [66].

It is important to point out that diaphragm contractile dysfunction from hypoxemia and hypercapnia creates positive feedback cycles exacerbating both conditions. This is because of fatigue-induced alveolar hypoventilation. In other words, diaphragm pump insufficiency caused by hypoxemia begets a further drop in blood oxygen levels. Ultimately, escalation of this unstable process could culminate in rapidly critical hypoxemia, causing sudden respiratory and cardiac arrests similar to the Hussain dog experiments [55]. This is also consistent with the features of many pediatric deaths in general, including unwitnessed ones like SIDS, given many are unexpected, sudden in onset, and rapid. Death by respiratory arrest would be silent too, another feature of SIDS.

Importantly, nicotine has a potent direct effect on skeletal muscles. In excess, even with minute ingestions in young children, death occurs rapidly, via rapid ventilatory muscle paralysis (again, by tetanic-like diaphragm arrest) [67]. In spontaneously breathing, anesthetized dogs oral and intravenous nicotine, peripheral respiratory arrests occurred in less than 15 min, causing death if not artificially ventilated [68]. Furthermore, ex vivo rabbit, frog, and cat limb muscles exposed to nicotine developed tetanic contractures [69–71]. Given that the diaphragm is also a skeletal muscle and high nicotine levels have been found in the tissues of SIDS victims [72], it is important to consider the potential for sudden diaphragm arrest (by contracture) to occur in infants exposed to tobacco smoke, especially in the presence of other diaphragm fatiguing DD factors. Nicotine could effectively lower the cramp threshold.

Evidence for of diaphragm myopathy in SIDS is provided by several histological studies [73, 76]. Focal, segmental, and diffuse diaphragm myofiber disruptions and contraction band necrosis, along with fibrotic scars in some near-miss SIDS cases were first reported by Kariks in 1989 [73]. Inflammatory cell infiltration (myositis) was not present in this study, suggesting a rapid, terminal onset of terminal changes. Contraction band necrosis (CBN), indicative of terminal asphyxia and anoxia, was confirmed in 82% of 242 SIDS cases in the this systematic study and later was corroborated by the three others. Importantly, despite the peculiar findings of “extreme compaction of sarcomeres in hypercontracted segments” in CBN, a mechanism has never been proposed. Research in this area inexplicably appears to have stalled. It also remains undetermined whether these myopathic changes might exist in sepsis and nicotine deaths. Regardless, given such compelling findings, it is reasonable to mandate diaphragm histology in all cases of sudden pediatric death, especially those with respiratory and / or gastrointestinal viral infections, bacterial infections, sepsis or nicotine exposure.

Further evidence of diaphragmatic histopathological changes during infection was provided in four other reports. The first, by Eisenhut (2011), documented focal infiltrates, myofiber destruction, and myocyte necrosis, and regeneration and in a 5-month-old with respiratory syncytial virus (RSV) infection, who had died in the hospital from sudden and unexpected respiratory arrest [76]. This was nearly identical to another paroxysmal respiratory arrest reported recently in an infant with RSV bronchiolitis [77]. Autopsy findings could not explain the death; however, diaphragm histology was omitted (not unusual because autopsy guidelines did not mandate this). A third report revealed, that three young children, aged 3 days to 5 years, who died unexpectedly from respiratory arrest, had exhibited a combination of diaphragm necrosis, inflammatory infiltration, and fiber regeneration at autopsy [78]. The 5-year-old previously healthy girl, who complained of chest and abdominal pain just before collapsing, had a hemoglobin level of 10.7 g/dL and a pH of 6.59. In addition to this extreme acidosis, the anemia could have contributed to diaphragm fatigue and failure because of the reduced blood oxygen-carrying capacity of blood. The fourth paper, a case‒control study examining the association of severe COVID-19 infection with the respiratory muscles of critically ill adult intensive care unit (ICU) patients, revealed direct viral infiltration of the diaphragm with fibrosis and regeneration in the case patients only [79].

Taken together, the above findings suggest that viral myositis and the myopathic changes in the diaphragms of young children with respiratory infections could have interfered with excitation–contraction coupling or electromechanical function of this organ, leading to escalating diaphragm fatigue and terminating in death by paroxysmal diaphragmatic failure. Because of the suddenness, it is possible such respiratory arrests may have occurred by critical hypoxemia-induced pathological diaphragm hyperexcitation, in the form of a rapid onset sustained cramp culminating in death by sudden respiratory arrest. This would explain the 5-year-old’s chest and abdominal pain prior to arresting. This could be the source of the hypercontraction injury and contraction band necrosis commonly seen in SIDS and would be consistent with the sustained contractions of putative diaphragm cramp-contracture.

By comparison, “myocardial electrical instability,” in the form of cardiac arrhythmias, was discussed in a 2019 summary report on viral myocarditis [80]. Sudden cardiac death, which is more common in males under 40 years of age, is particularly concerning because the incidence of occult myocarditis at autopsy was as high as 44%. Moreover, infectious myositis of the myocardium in sudden unexpected deaths is more common than thought with the same occurring in the diaphragm leading to the same fatal outcome.

### Metabolic acidosis, diaphragmatic failure and lethal arrhythmias

Sepsis disrupts acid‒base balance via a variety of mechanisms leading to metabolic acidosis. This fatigues skeletal muscles of the limbs, trunk and diaphragm, which is particularly aggravated by hypoxemia and hypercapnia [81, 82]. In critically ill patients, metabolic acidosis generally occurs through the accumulation of acidic anaerobic metabolites, which are increased by cytokines and other inflammatory mediators during infection, as well as bicarbonate loss in severe diarrhea or renal insufficiency. It is often accompanied by electrolyte imbalances resulting from fluid loss and disrupted renal function. Tissue hypoperfusion with reduced lactate clearance by the liver and kidneys leads to lactic acidosis, the primary cause of inpatient metabolic acidosis. Elevated lactate levels serve as a prognostic marker of disease severity and mortality. Although the causal relationship is unknown, decompensation of comorbid conditions, vascular smooth muscle dysfunction, myocardial depression and cardiac arrhythmias are most often cited [83]. Less is known about respiratory failure because it is often concealed, as these severely ill patients are already receiving lifesaving mechanical ventilation.

Extracellular pH has a major influence on skeletal (and heart muscle) electrophysiology, similar to the mechanisms already described in sepsis, leading to pathological inexcitability (atrophy and fatigue) and hyperexcitation. As alluded to above, ventricular fibrillation (VFib) can be considered a form of muscle cramp. Its quivering, arrhythmic and ineffective contractions significantly impair cardiac output, causing pump failure. This malignant arrhythmia is sensitive to the acid‒base balance of blood. In anesthetized dogs, infusion of organic acids from a physiologic pH of 7.42 to 7.21 progressively lowered the threshold for VFib [84] (which was reversed by alkaline infusion) [84]. This could have occurred through an altered conductance of voltage-gated ion channels, for example, the *hERG1* potassium channel (expressed in the heart) [85] or the *SCN5A* sodium channel (heart and skeletal muscles) [86]. The latter plays a critical role in physiological excitation but is extremely sensitive to low pH [87]. Both channelopathies have been suspected to cause sudden unexpected deaths by inducing cardiac arrhythmias, primarily long QT syndrome. Similarly, unstable diaphragmatic arrhythmias may also be triggered. Unfortunately, however, this organ has been entirely omitted from ion channel tissue distribution studies. This might explain why only 2% of 93 SIDS victims had a *SCN5A* channel defect in their myocardia (and not any higher) [88]. In other words, a higher prevalence might have existed had their diaphragms been examined which illustrates restricted thinking in mainstream research.

Metabolic acidosis in young children with inborn errors of metabolism has also been reported in association with sudden deaths (or near-deaths). Some of these disorders involve defects of mitochondrial electron transport proteins causing cardiomyopathy and skeletal muscle myopathy (e.g., myalgia, hypotonia and fatigue) [89, 90]. In these case reports, sudden respiratory distress, including apnea and labored, agonal breathing, occurred prior to cardiopulmonary arrests. Interestingly, RSV and rotavirus infections were noted in addition to severe acidosis, some with copious vomiting and diarrhea. Given this and the associated myopathies presumably involving the diaphragm, peripheral respiratory failure could have been responsible for the deaths/near-deaths.

Pediatric deaths from severe diarrheal illness in developing countries, which also occur rapidly and unexpectedly, are often associated with bicarbonate-loss -induced hyperchloremic acidosis (as well as hyponatremia and hypokalemia) [91, 92]. Among patients seen in hospitals, terminal pathological mechanisms include VFib and rapidly progressive respiratory distress; however, there is a paucity of information on the latter other than “complications of respiratory muscle fatigue” (again, obscured by mechanical ventilation). With metabolic acidosis, the fatigue manifests in response to CNS-mediated Kussmaul’s respirations: compensatory tachypnea and hyperpneas to “blow off” CO_2_, that can deteriorate in extremis to agonal breathing. Similarly, this occurs in diabetic ketoacidosis, where many deaths are also sudden and unexpected, even in the hospital setting [93]. pH is extremely low and associated with a hyperosmolar state featuring extremely elevated lactate levels, hypokalemia, hypomagnesemia, and hypophosphatemia. In a retrospective observational case study involving patients of all ages, including infants, at least 30 of 69 died by witnessed sudden respiratory arrest (terminal apnea). Most, but not all these respiratory arrests, were preceded by mental status changes suggestive of terminal cerebral edema (thought to cause central respiratory arrest). However, in 20 patients, including most infants and toddlers, there was no change before the respiratory arrest. This suggests the deaths may not have been centrally induced and is consistent with other authors’ conclusions [94]. Given that increased work of breathing occurs in severe acidosis and that the resulting diaphragm fatigue is exacerbated by concomitant hypovolemia, hypoxemia, hypercapnia, and electrolyte disorders, it is reasonable to propose that peripheral respiratory failure, caused by sudden diaphragmatic arrest, could be a terminal pathological mechanism in severe acidosis.

The mechanisms leading to skeletal muscle dysfunction in metabolic (and respiratory) acidosis are quite complex. Essentially, intracellular acidosis increases ionized calcium bound within sarcoplasmic reticulum (SR) stores and reduces its SR uptake. The lack of available SR calcium disrupts excitation‒contraction coupling, leading to muscle fatigue by both a reduction in contractile force and prolongation of the muscle relaxation phase, a process that is load sensitive (i.e., the heavier the workload is, the less muscle shortening and the longer the relaxation phase) [95–97]. This is known as negative lusitropy and is mirrored by cardiac diastolic dysfunction. Under higher heart rates, the dysfunction is exacerbated by a delay in left ventricular relaxation, leading to reduced cardiac output. This is manifested by worsening exercise tolerance and congestive heart failure [98] and puts heart failure patients at risk for sudden arrhythmias and cardiac arrest [99].

In terms of the diaphragm, ex vivo studies have revealed that fatigue-induced tetanic contracture develops when the relaxation time is excessively prolonged [59]. This is exacerbated by acidosis [97], endotoxins [54] and fatigue itself. (Notably, DD improves with methylxanthines such as theophylline and caffeine, which have been used for over 50 years to treat apnea and periodic breathing in preterm infants [100].) In vivo, this delay could be problematic under higher-frequency breathing: when relaxation takes longer than does the expiratory phase of the respiratory cycle, incomplete return to the resting position occurs. Consequent air trapping (breath stacking) and hyperinflation would reduce the mechanical advantage at the diaphragm, thereby exacerbating the DD. Furthermore, given that diaphragmatic perfusion occurs primarily during the relaxation phase, higher-frequency breathing could then lead to metabolic mismatch within the organ [96], thereby contributing to further fatigue in another DD positive feedback cycle (in addition to the hypoxemic‒hypercapnic one already discussed). With worsening fatigue by dehydration, acidosis, endotoxins, electrolyte disorders, and myopathy, the relaxation delay could explain pathological excitation. If it occurs as transient diaphragm spasm apneas, the ensuing hypoxemia would be survivable. However, when sustained as a cramp-contracture respiratory arrest, the severe hypoxemia could be fatal (by secondary cardiac arrest).

Importantly, in vitro experiments have shown that lactic acidosis decreases diaphragmatic contractility but only at very low “extraphysiological” pH values of 6.80 [101]. However, McGaffey’s report of an average pH of 6.15 in forty SIDS victims suggests otherwise. Thus, extrapolating in vitro data to in vivo animal or human studies requires special caution (vide infra). Kimmoun et al. found that no survival has been reported for severe lactic acidosis with shock under pH 7.0 [83].

Although human sepsis causes metabolic acidosis and can result in cardiac and diaphragmatic failure, animal experiments in rats have been unsuccessful in demonstrating this adverse outcome, with respiratory acidosis causing diaphragm failure but metabolic acidosis not [102]. As alluded to above, limitations in animal and/or in vitro experimental conditions make extrapolation to the human model imprudent.

### Other causes of vital-organ failure in *sepsis*

The other causes mentioned above, namely cardiac arrhythmias/asystole with increased risk from genetic predisposition, cardiac asystole secondary to sepsis-induced hyperkalemia [103–105], respiratory failure (CNS-based) [106] and genetically predisposed mitochondrial energy failure (inborn errors of metabolism) [107], have been reviewed elsewhere and are, therefore, not included in this paper. However, it is noteworthy that while “infection” might be listed as an accompaniment, mention of sepsis is rare, if it is ever made, in articles on inborn errors of metabolism and SIDS.

## Conclusions

The finding of extreme metabolic acidosis and extreme hyperkalemia in SIDS in several relatively obscure reports has important implications for an improved understanding of SIDS pathogenesis. Given that infection strongly correlates with all SIDS risk factors, this logically leaves sepsis/septic shock as the common link in generating conditions that lead to metabolic acidosis. Vital end-organ dysfunction and outright failure may result from pathogen and host reactions, as well as the pathological effects of various associated humoral biproducts and metabolite‒electrolyte changes. The evidence for a connection between metabolic acidosis and SIDS uniquely revealed in this review is compelling and provides a solid basis for further work to define which pathway (or pathways), leads to the tragedy of SIDS. This review provides a basis for incorporating pH, lactate and electrolyte levels, as well as diaphragmatic histopathology into formal autopsy protocols when investigating sudden infant deaths. Many protocols recommend these investigations but are often not followed [108]. Intensified research into metabolic acidosis in SIDS is clearly warranted.

## Data Availability

Data sharing not applicable to this article as no datasets were generated or analysed during the current study.
